# Supplementary material for: Metabolic Response After a Single Maximal Exercise Session in Physically Inactive Young Adults (EASY Study): Relevancy of Adiponectin Isoforms
Source: Biomolecules. 2025 Feb 20;15(3):314. doi: 10.3390/biom15030314 (PMC11940768; doi:10.3390/biom15030314)

Supplementary material

Metabolic response after a single maximal exercise session in physically inactive young adults (EASY-Study). Relevancy of adiponectin isoforms.

Representative blot of adiponectin

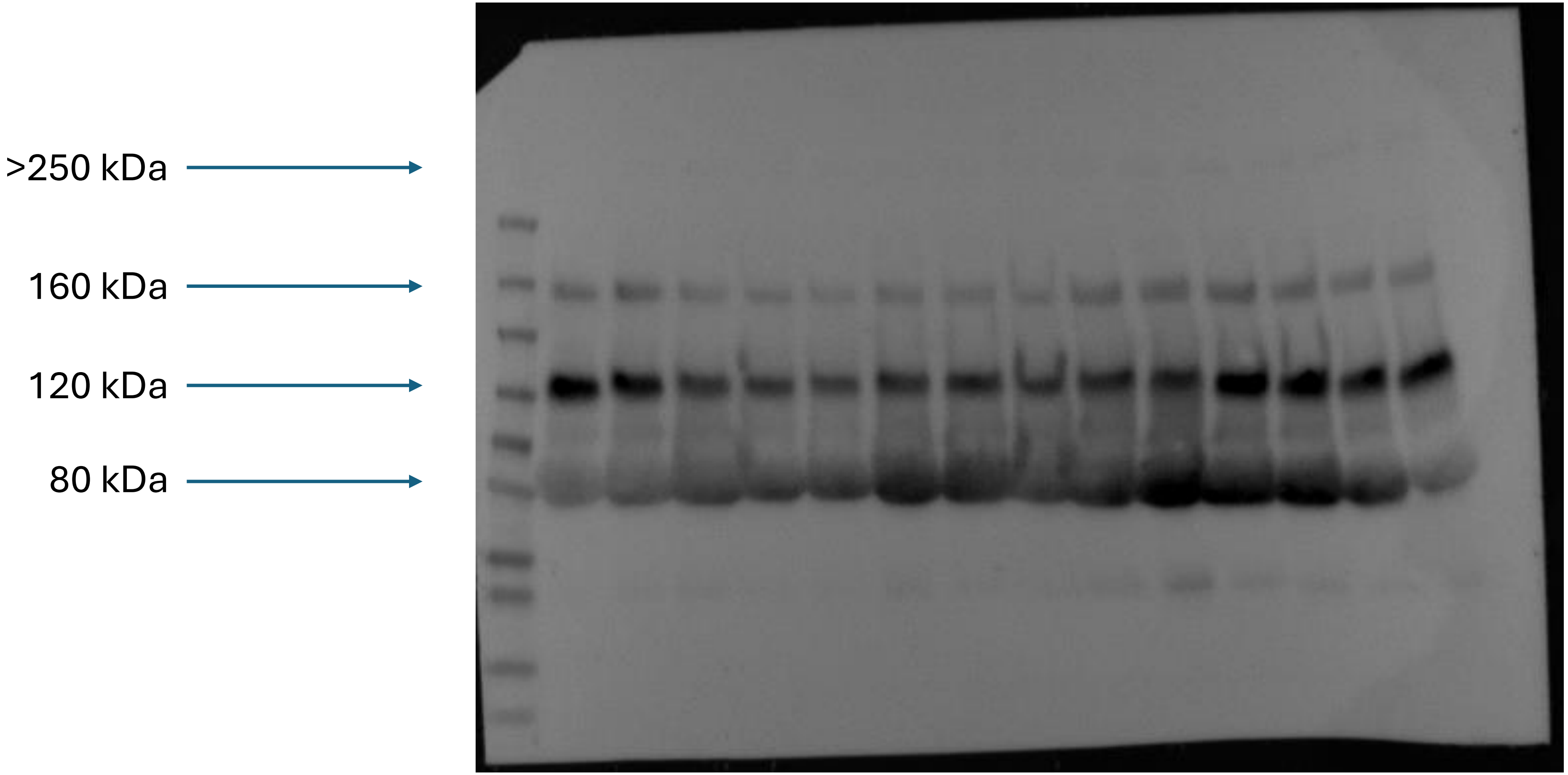

Representative blot with Ponceau S

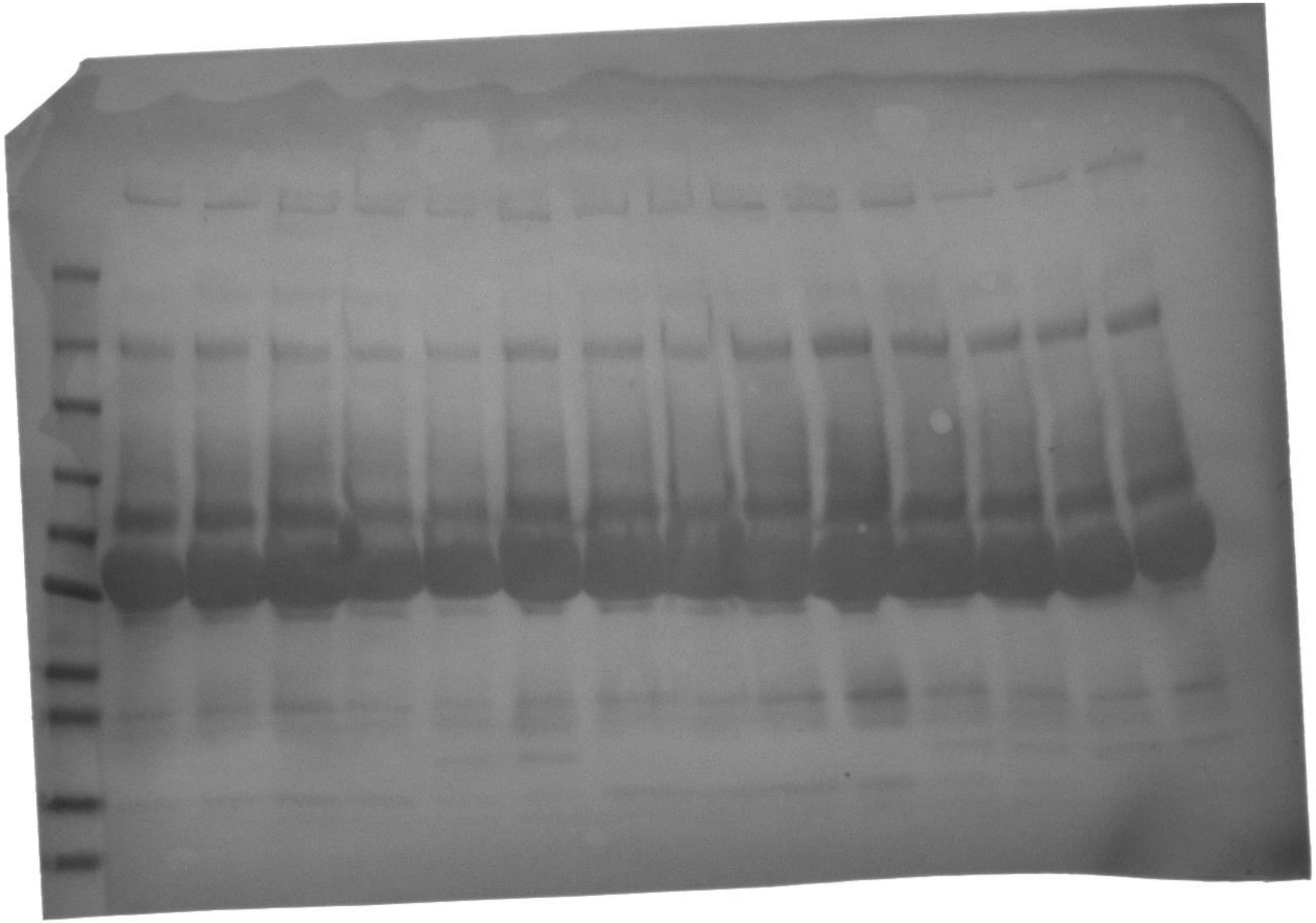

Supplement: Supplementary file 1 [file biomolecules-15-00314-s001.zip › biomolecules-3465393-supplementary.pdf]
